# Impact of Sex on Clinical Outcomes of Tandem Occlusion in Acute Ischemic Stroke Patients Treated With Mechanical Thrombectomy. A Propensity‐Matched Analysis

**DOI:** 10.1111/ene.70044

**Published:** 2025-01-13

**Authors:** Lucio D'Anna, Matteo Foschi, Mariarosaria Valente, Liqun Zhang, Simona Sacco, Raffaele Ornello, Nina Mansoor, Matthew Fallon, Adelaida Gartner Jaramillo, Massimo Sponza, Vladimir Gavrilovic, Kyriakos Lobotesis, Gian Luigi Gigli, Soma Banerjee, Giovanni Merlino

**Affiliations:** ^1^ Department of Stroke and Neuroscience, Charing Cross Hospital Imperial College London NHS Healthcare Trust London UK; ^2^ Department of Brain Sciences Imperial College London London UK; ^3^ Department of Biotechnological and Applied Clinical Sciences University of L'Aquila L'Aquila Italy; ^4^ Clinical Neurology, Udine University Hospital and DMED University of Udine Udine Italy; ^5^ Department of Neuroscience George's University of London, Stroke London UK; ^6^ Neuroradiology Udine University Hospital Udine Italy; ^7^ Vascular and Interventional Radiology Udine University Hospital Udine Italy; ^8^ Neuroradiology, Department of Imaging, Charing Cross Hospital Imperial College London, NHS Healthcare Trust London UK; ^9^ Stroke Unit Udine University Hospital Udine Italy; ^10^ Clinical Neurology, Udine University Hospital and DAME University of Udine Udine Italy

**Keywords:** acute ischemic stroke, mechanical thrombectomy, sex, tandem, tandem occlusion

## Abstract

**Background:**

Although mechanical thrombectomy (MT) represents the standard of care for ischemic stroke due to large‐vessel occlusion (LVO), the impact of sex on outcomes in tandem occlusions remains unclear. We investigated sex‐based differences in outcomes after MT for tandem occlusions.

**Methods:**

This multicenter observational study included consecutive patients with tandem occlusion treated with MT across three stroke centers (2021–2023). Propensity score matching was performed. Primary outcomes were the 90‐day favorable functional outcome (mRS 0–2) and mRS score shift. Secondary outcomes included favorable recanalization, 24‐h early neurological improvement, and NIHSS median score. Safety outcomes were post‐MT intracerebral hemorrhage and 90‐day mortality.

**Results:**

Of 635 patients (46.8% women), 289 women were matched to 289 men. There were no significant differences in primary, secondary, or safety outcomes between sexes. Subgroup analysis showed a lower rate of favorable 90‐day mRS scores in women with diabetes compared to men. Women not receiving emergent carotid treatment had higher rates of favourable outcomes. No significant sex differences were found in other subgroups.

**Conclusions:**

Women with anterior circulation tandem occlusions treated with MT have similar outcomes to men. However, women with diabetes and those treated with intracranial MT alone exhibited sex‐specific differences. Further studies are needed to explore underlying mechanisms.

## 1 Introduction

Tandem occlusions consist in the presence of occlusion, sub‐occlusion, or stenosis of the extracranial internal carotid artery (ICA), due to atheromatous plaque or dissection, together with simultaneous intracranial large‐vessel occlusion [1]. Mechanical thrombectomy (MT) currently represents the standard of care for acute ischemic stroke due to large‐vessel occlusion (LVO) even in acute ischemic strokes due to anterior circulation tandem occlusions, as treatment with MT is associated with higher odds of a favorable outcome compared with medical therapy alone [2]. Nevertheless, the presence of an ICA steno‐occlusive lesion poses technical challenges that interventional neuroradiologists face while treating tandem occlusions with MT. These types of occlusions are present in up to one‐third of all patients with stroke undergoing MT and are associated with worse clinical outcomes [3, 4]. Two treatment strategies have been used in clinical trials at the discretion of the operators: acute ICA stenting plus antithrombotic therapy or intracranial thrombectomy alone [5]. Extracranial ICA angioplasty is often implemented alongside these treatment modalities [6]. However, the lack of randomized trials and the conflicting results from observational studies have left several unanswered questions regarding the best management of tandem occlusions, as highlighted in a recent international survey regarding acute treatment options [7] for tandem occlusions.

The impact of sex differences in outcomes in patients with acute ischemic stroke due to anterior circulation LVO undergoing MT is still unclear, especially in patients with tandem occlusion [8]. Indeed, previous studies reported conflicting results regarding the impact of sex on clinical outcomes after MT for anterior circulation tandem occlusion [9, 10, 11]. As women live longer, they are usually older when they have their first stroke. However, despite an increasing rate of inclusion of women in global registries and trials in more recent years [12], women are still underrepresented in stroke trials [13, 14], therefore limiting the value of the available evidence from observational studies and registries.

In this study, we investigated the impact of sex differences on the outcomes of patients with anterior circulation tandem occlusions undergoing MT across three comprehensive stroke centers.

## 2 Methods

This is a multicentre, observational, investigator‐initiated, post hoc analysis from prospective collected data from local registries, that included all acute stroke patients aged 18 years or older with anterior circulation tandem occlusion due to atherosclerosis or dissection consecutively treated with MT in three thrombectomy capable centers: Charing Cross Hospital, Imperial College Healthcare NHS Trust, London (UK); St George's University of London, London (UK); Udine University Hospital, Udine (Italy) between January 1, 2021 and March 30, 2023 with local stroke registries available [15, 16, 17]. Tandem lesion was defined as a proximal intracranial occlusion (intracranial occlusion of internal carotid artery [ICA] or of M1‐M2 segments of the middle cerebral artery [MCA] and a cervical occlusion or stenosis ≥ 50%). All patients underwent computed tomography (CT) of brain including CT angiography or magnetic resonance imaging (MRI) including MRI angiography. Intravenous thrombolysis (IVT) with intravenous tissue plasminogen activator (tPA) was administered in all patients who presented within 4.5 h of stroke symptom onset and without contraindications according to current guidelines [18]. All patients received MT of the intracranial occlusion with and without cervical ICA lesion intervention. The cervical ICA treatment approach was based on the operator's preference based on patient vessel anatomy, clinical status, and operator experience. All patients who underwent carotid stenting received antiplatelet therapy immediately after the procedure, with no differences between sexes, in accordance with each center's protocol. Both intracranial and extracranial occlusions were treated emergently at the same MT session. There was no standardized selection protocol for MT treatment, and patients were selected for MT based on the participating centre protocol. Ethical approval for this study was obtained from the institutional review boards of all participating centres. Given the retrospective nature of the study and the use of fully anonymized data collected as part of routine clinical care, informed consent was waived in accordance with local regulations. All data were handled in compliance with data privacy and protection standards, ensuring confidentiality and adherence to ethical guidelines.

For the purpose of this analysis, the criteria for patient selection were (1) age ≥ 18 years; (2) National Institutes of Health Stroke Scale (NIHSS) score of 6 or more; (3) Alberta Stroke Program Early CT score (ASPECTS) 5 or more [19]; (4) Intracranial occlusion of the ICA or occlusion of the M1–M2 segments of the MCA and a cervical occlusion or stenosis ≥ 50%; (5) initiation of MT had to be possible within 6 h after stroke onset; (6) pre‐event modified Rankin scale (mRS) score of 0–2.

### 2.1 Propensity Score Matching

We calculated propensity scores for each patient using a multivariable logistic regression model that included demographics (age), risk factors (hypertension, diabetes, hyperlipidemia, smoking status, atrial fibrillation, and prior TIA/ischemic stroke), pre‐stroke functional status (mRS), pre‐thrombectomy Alberta Stroke Scale Early CT Score (ASPECTS), large‐vessel occlusion characteristics (degree of extracranial carotid stenosis, site of distal occlusion), stroke severity (NIHSS at admission), and procedural features (onset to groin time, administration of intravenous thrombolysis, type of anesthesia, type of technique for distal thrombus thrombectomy, type of emergent carotid treatment, intraprocedural use of heparin, number of passes, and periprocedural use of antithrombotic). Female patients were matched with male patients in a 1:1 ratio, within 0.2 standard deviations of the logit of the propensity score, using greedy nearest neighbor matching. The quality of the matching was assessed by comparing the standardized difference of means and the ratio of variances between the propensity scores of treatment groups, as well as by inspecting graphical distributions of propensity scores and covariate balance between the matched cohorts.

### 2.2 Outcomes

The primary effectiveness outcome were the 90‐day favorable functional outcome, as defined by a modified Rankin scale score of 0–2, and the 90‐day shift in patient mRS scores between women and men. The secondary effectiveness outcomes included favorable recanalization (post‐procedural Treatment in Cerebral Infarction [TICI] score of 2b, 2c or 3), post‐MT ordinal distribution of TICI scores, 24‐h early neurological improvement and deterioration (≥ 2 points decrease or increase in the NIHSS score from baseline, respectively), 24‐h median change in the NIHSS score from baseline. The safety outcomes were post‐MT any intracerebral hemorrhage (ICH), as defined by any category of the Heidelberg bleeding classification system, post‐MT ordinal distribution of ICH categories, post‐MT symptomatic hemorrhage (any ICH causing ≥ 4 points worsening on the NIHSS score), 90‐day all‐cause mortality. The occurrence of all outcomes was adjudicated by local investigators and validated by an expert neuroradiologist (for outcomes involving neuroimaging exam).

### 2.3 Statistical Analysis

Categorical variables were reported as number and percentage, continuous variables as mean and SD or median and interquartile range (IQR), according to their distribution. For the primary effectiveness outcomes, we calculated the risk ratio and risk difference with 95% confidence intervals (CIs) for the 90‐day occurrence of favorable mRS score between women and men. The 90‐day shift of mRS scores was compared using an ordinal generalized linear model (GLM) and results were presented as odds ratio (OR) with 95% CIs. We applied a proportional odds model (cumulative logit), which is appropriate for the ordinal nature of the mRS categories. This method allows for a clinically meaningful interpretation of treatment effects across the entire mRS scale while preserving the rank order of disability outcomes. For the secondary effectiveness outcomes, risk ratios and differences between sexes were calculated for the occurrence of post‐procedural favorable TICI score, 24‐h early neurological improvement and deterioration. Changes in the 24‐h NIHSS scores from baseline between women and men were compared using linear regression, and results were presented as OR and 95% CIs. The ordinal distribution of post‐procedural TICI score was compared using Chi‐square test. For all the safety outcomes, we also calculated risk differences and risk ratios with 95% CIs between the sex groups, while the 90‐day ordinal distribution of ICH categories was compared with chi‐squared test. All outcomes analyses were performed separately in the unmatched and matched cohorts. Subgroup analysis of the primary effectiveness outcome in the matched cohort was performed calculating sex differences in 12 prespecified subgroups (age [< 50 years or ≥ 50 years and < 65 years or ≥ 65 years], NIHSS score at onset [0 to 5, 6 to 10, 11 to 42], ASPECTS [< 6 or ≥ 6], hypertension, diabetes, atrial fibrillation, site of distal occlusion [carotid T, ICA + M1, ICA + M2], intravenous thrombolysis, onset to groin time [≤ 360 min, > 360 min], type of anesthesia [general or local], thrombectomy technique for distal thrombus [stenting, aspiration, combined], treatment of emergent carotid [none, balloon angioplasty plus stenting, balloon angioplasty alone]). Lastly, we used a GLM model with sex, subgroup variable, and their interaction term as independent variables to assess the homogeneity in the effect of sex by each subgroup variable. The *p* value was presented for the interaction term. As the outcomes of the present study were exploratory and there were no assumptions regarding the propensity score matching, a sample size was not prespecified for this analysis. All statistical analyses were performed using R software, version 4.2. Statistical significance was set at a *p* value < 0.05.

## 3 Results

**FIGURE 1:**
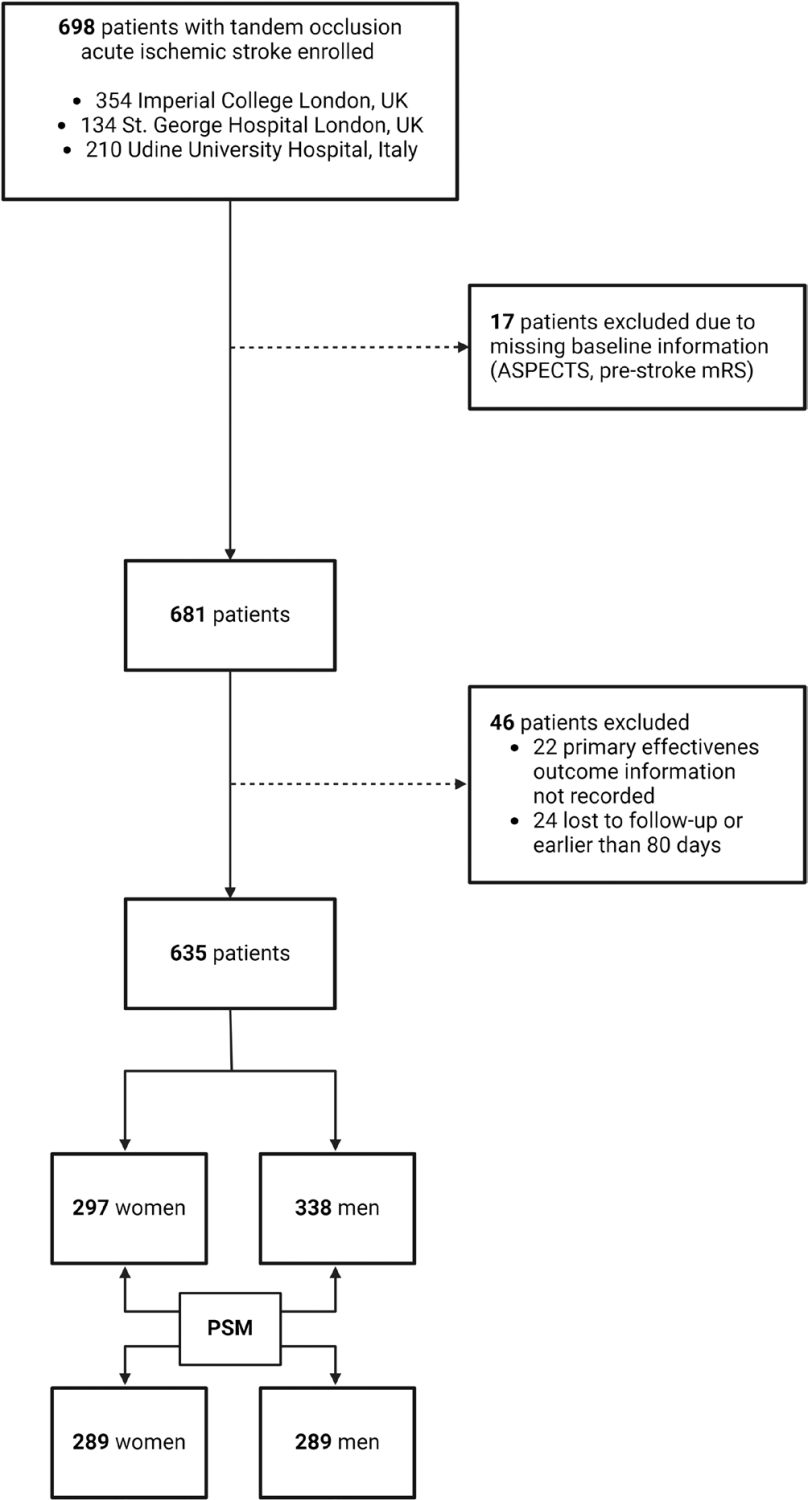
Study flowchart. ASPECTS, Alberta Stroke Program Early CT score; mRS, modified Rankin score; PSM, propensity score matching.

Overall, a total of 635 patients were included in the analysis, of whom 297 (46.8%) were women. The number of excluded patients with reasons is reported in the study flowchart (Figure 1). Within the unmatched cohort (Table 1), women had a different distribution of the site of the distal large‐vessel occlusion (*p* = 0.002) compared to men. However, distribution of the other study variables was similar between sex groups. We paired 289 women and 289 men with tandem occlusion treated with MT. No missing data were recorded in the variables used to calculate the propensity scores. Measures of balance diagnosis indicated that the samples were adequately matched, with a standardized difference of the propensity scores means between groups of 0.12 (good balance < 0.25), ratio of variances of propensity scores 1.09 (good balance between 0.5 and 2). Graphics of propensity scores and covariates balance distributions confirmed a good overall quality of the matching (Figures S1 and S2).

**TABLE 1:** Baseline characteristics of the unmatched and matched cohorts.

	Unmatched cohort				Matched cohort			
	Women (*n* = 297)	Men (*n* = 338)	Standardized difference*	*p*	Women (*n* = 289)	Men (*n* = 289)	Standardized difference*	*p*
*Demographics*								
Age, median (IQR)	69 (56–80)	70 (58–80)	0.042	0.750	69 (56–80)	70 (57–80)	0.031	0.838
*Clinical characteristics*								
NIHSS on admission, median (IQR)	17 (12–21)	17 (10–20)	0.115	0.152	17 (12–21)	18 (12–21)	0.001	0.975
mRS score category, *n* (%)			0.092	0.511			0.077	0.653
No symptoms (score of 0), *n* (%)	209 (70.4)	251 (74.3)			202 (69.9)	212 (73.4)		
Symptoms without any disability (score of 1), *n* (%)	63 (21.2)	60 (17.8)			62 (21.5)	55 (19.0)		
Symptoms with mild disability (score of 2), *n* (%)	25 (8.4)	27 (7.9)			25 (8.6)	22 (7.6)		
ASPECTS, median (IQR)	8 (7–10)	9 (7–10)	0.009	0.847	8 (7–10)	8 (7–10)	0.001	0.916
Site of distal occlusion, *n* (%)^a^			0.314	**0.002**			0.010	0.856
ICA‐T	34 (11.4)	36 (10.6)			34 (11.8)	35 (12.1)		
ICA + M1	247 (83.2)	253 (74.9)			239 (82.7)	235 (81.3)		
ICA + M2	16 (5.4)	49 (14.5)			16 (5.5)	19 (6.6)		
*Degree of extracranial stenosis, n (%)*								
50%–74%	13 (4.4)	13 (3.8)			12 (4.2)	11 (3.8)		
57%–99%	36 (12.1)	36 (10.65)			33 (11.4)	31 (10.7)		
100% (occlusion)	248 (83.5)	289 (85.5)			244 (84.4)	247 (85.5)		
*Pre‐stroke risk factors*								
Hypertension, *n* (%)^a^	151 (50.8)	184 (54.4)	0.072	0.409	147 (50.9)	149 (51.6)	0.014	0.934
Hypercholesterolemia, *n* (%)^b^	94 (31.6)	106 (31.4)	0.006	> 0.999	92 (31.8)	88 (30.4)	0.030	0.788
Diabetes mellitus, *n* (%)^c^	55 (18.5)	68 (20.1)	0.041	0.683	53 (18.3)	55 (19.0)	0.018	0.915
Current smoking, *n* (%)^d^	73 (24.6)	105 (31.1)	0.145	0.085	72 (24.9)	81 (28.0)	0.071	0.451
Previous TIA/ischemic stroke, *n* (%)	34 (11.4)	38 (11.2)	0.006	> 0.999	34 (11.8)	34 (11.8)	< 0.001	> 0.999
Atrial fibrillation, *n* (%)	80 (26.9)	84 (24.9)	0.048	0.612	78 (27.0)	73 (25.3)	0.039	0.715
*Therapy on admission, n (%)*								
Oral anticoagulation, *n* (%)	38 (12.8)	39 (11.5)	0.038	0.717	37 (12.8)	33 (11.4)	0.042	0.702
Antiplatelet treatment, *n* (%)	40 (13.5)	63 (18.6)	0.141	0.100	39 (13.5)	52 (18.0)	0.124	0.171
*Procedural features, n (%)*								
Intravenous thrombolysis, *n* (%)	188 (63.3)	214 (63.3)	< 0.001	> 0.999	181 (62.6)	185 (64.0)	0.029	0.796
Onset to needle time (min), median (IQR)**	144 (110–200)	144 (110–200)	0.044	0.906	144 (110–200)	135 (110–196)	0.108	0.381
Onset to groin time (min), median (IQR)	271 (110–374)	273 (110–370)	0.071	0.898	272 (110–372)	271 (110–374)	0.010	0.647
Type of anesthesia, *n* (%)								
General	242 (81.5)	275 (81.4)	0.003	> 0.999	236 (81.7)	235 (81.3)	0.010	> 0.999
Local	55 (18.5)	63 (18.6)			53 (18.3)	54 (18.7)		
Distal thrombus thrombectomy technique, *n* (%)								
Stent retriever	11 (3.7)	20 (5.9)	0.117	0.347	11 (3.8)	13 (4.5)	0.050	0.833
Aspiration	155 (52.2)	181 (53.6)			153 (53.0)	157 (54.3)		
Combined	131 (44.1)	137 (40.5)			125 (43.2)	119 (41.2)		
Treatment of emergent carotid, *n* (%)								
None	228 (76.8)	253 (74.9)	0.076	0.637	222 (76.8)	220 (76.1)	0.059	0.777
Balloon angioplasty plus stenting	49 (16.5)	65 (19.2)			47 (16.3)	52 (18.0)		
Balloon angioplasty alone	20 (6.7)	20 (5.9)			20 (6.9)	17 (5.9)		
Number of passes, median (IQR)	3 (2–4)	2 (1–4)	0.033	0.160	3 (2–4)	2 (1–4)	0.055	0.892
Intraprocedural heparin, *n* (%)	11 (3.7)	19 (5.6)	0.091	0.343	11 (3.8)	13 (4.5)	0.035	0.835
Periprocedural antithrombotic, *n* (%)	57 (19.2)	88 (26.0)	0.164	0.051	57 (19.7)	63 (21.8)	0.051	0.608

*Note:* Statistically significant *p* values (< 0.05) are reported in bold.

Abbreviations: ASPECTS, Alberta Stroke Program Early CT score; ICA, internal carotid artery; IQR, interquartile range; M, middle cerebral artery; mRS, modified Rankin Score; NIHSS, National Institutes of Health Stroke Scale; T, carotid T occlusion.

^a^ Hypertension was defined as a history of blood pressure > 140/90 mmHg and/or the current use of antihypertensive medications.

^b^ Hyperlipidemia was defined as a history of total blood cholesterol levels > 220 mg/dL and/or the current use of lipid‐lowering drugs.

^c^ Diabetes mellitus was defined as a history of fasting glucose > 126 mg/dL or the current use of hypoglycemic medications.

^d^ Current smoking was defined as the consumption of ≥ 1 cigarette per day over the last year.

* A standardized difference (of means) < 0.250 indicates that the groups are well balanced.

** Only patients who underwent intravenous thrombolysis.

The comparison of baseline characteristics further supported the good balance of our matched cohorts, with a standardized difference of the propensity scores < 0.25 in all variables (Table 1).

### 3.1 Primary Effectiveness Outcome

**FIGURE 2:**
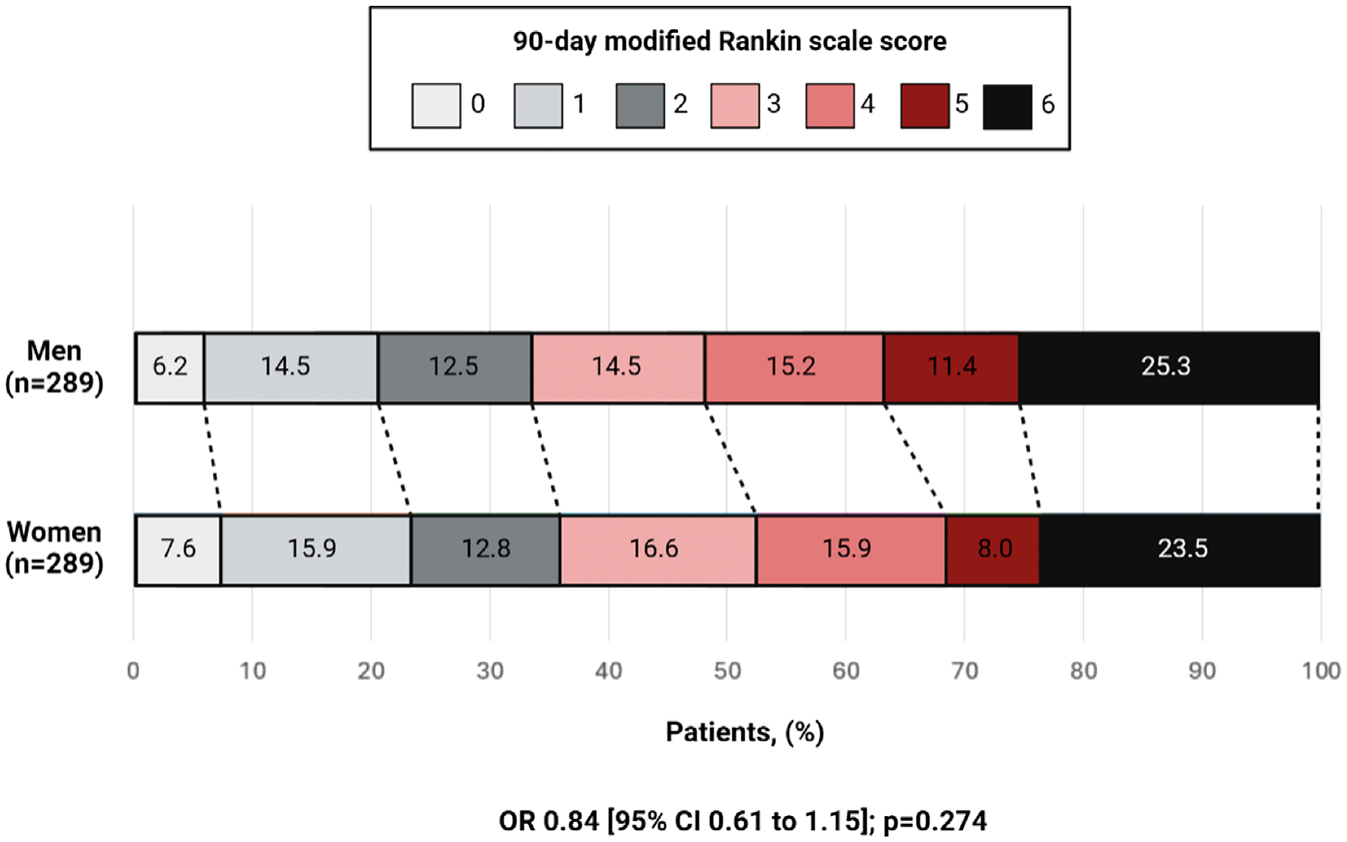
Ordinal mRS distribution between sexes in the matched cohort.

We did not find a significant difference in the rate of favorable 90‐day mRS score (0–2) (RR 0.95 [95% CI 0.77 to 1.17]; *p* = 0.623) and 90‐day mRS distribution (OR 1.00 [95% CI 0.73 to 1.37]; *p* = 0.980) between women and men in the unmatched cohort (Table 2). After propensity score matching, the rate of favorable 90‐day mRS score (0–2) (RR 1.07 [95% CI 0.86 to 1.34]; *p* = 0.541) and the 90‐day mRS distribution (OR 0.84 [95% CI 0.61 to 1.15]; *p* = 0.274) were similar between sexes. Moreover, in the matched cohort the mRS shift analysis did not demonstrate a significant difference in the 90‐day ordinal distribution of mRS scores between women and men (Figure 2).

**TABLE 2:** Outcomes comparison in the unmatched and matched cohorts.

	Unmatched cohort		Matched cohort		Statistical metric	Unmatched cohort		Matched cohort	
	Women (*n* = 297)	Men (*n* = 338)	Women (*n* = 289)	Men (*n* = 289)		Treatment difference [95% CI]	*p*	Treatment difference [95% CI]	*p*
*Primary effectiveness outcomes*									
90‐day favorable mRS score (0–2), *n* (%)	106 (35.7)	127 (37.6)	104 (36.0)	97 (33.6)	Risk ratio	0.95 [0.77 to 1.17]	0.623	1.07 [0.86 to 1.34]	0.541
					Risk difference (%)	−1.8 [−9.4 to 0.6]	0.623	2.4 [−5.3 to 10.2]	0.541
90‐day mRS score distribution					Odds ratio	1.00 [0.73 to 1.37]	0.980	0.84 [0.61 to 1.15]	0.274
No symptoms (score of 0), *n* (%)	22 (7.4)	27 (8.0)	22 (7.6)	18 (6.2)					
Symptoms without any disability (score of 1), *n* (%)	46 (15.5)	58 (17.2)	46 (15.9)	42 (14.5)					
Symptoms with mild disability (score of 2), *n* (%)	38 (12.8)	42 (12.4)	36 (12.5)	37 (12.8)					
Symptoms with mild‐to‐moderate disability (score of 3), *n* (%)	49 (16.5)	44 (13.0)	48 (16.6)	42 (14.5)					
Symptoms with moderate‐to‐severe disability (score of 4), *n* (%)	47 (15.8)	50 (14.8)	46 (15.9)	44 (15.2)					
Symptoms with severe disability (score of 5), *n* (%)	25 (8.4)	37 (10.9)	23 (8.0)	33 (11.4)					
Death (score of 6), *n* (%)	70 (23.5)	80 (23.7)	68 (23.5)	73 (25.3)					
*Secondary effectiveness outcomes*									
Post‐procedural favorable TICI score, *n* (%)	265 (89.2)	303 (89.6)	257 (88.9)	257 (88.9)	Risk ratio	1.00 [0.94 to 1.05]	0.864	1.00 [0.94 to 1.06]	> 0.999
					Risk difference (%)	−0.4 [−5.2 to 4.4]	0.864	0.0 [−5.1 to 5.1]	> 0.999
Post‐procedural TICI score distribution^a^					Chi‐square	—	0.723	—	0.763
0, *n* (%)	16 (5.4)	14 (4.1)	16 (5.5)	13 (4.5)					
1, *n* (%)	2 (0.7)	5 (1.5)	2 (0.7)	5 (1.7)					
2a, *n* (%)	14 (4.7)	16 (4.7)	14 (4.8)	14 (4.8)					
2b, *n* (%)	126 (42.4)	140 (41.4)	122 (42.2)	120 (41.5)					
2c, *n* (%)	18 (6.1)	28 (8.3)	18 (6.2)	23 (8.0)					
3, *n* (%)	121 (40.7)	135 (39.9)	117 (40.5)	114 (39.4)					
24‐h change in NIHSS score from baseline, median (IQR)	−3 (−8 to −1)	−3 (−8 to 0)	−3 (−8 to −1)	−3 (−8 to 0)	Odds ratio	1.13 [0.25 to 4.99]	0.874	0.81 [0.17 to 3.97]	0.803
24‐h early neurological improvement, *n* (%)	71 (23.9)	75 (22.2)	66 (22.8)	67 (23.2)	Risk ratio	1.08 [0.81 to 1.43]	0.608	0.99 [0.73 to 1.33]	0.921
					Risk difference (%)	1.7 [−4.9 to 8.3]	0.609	−0.3 [−7.2 to 6.5]	0.921
24‐h early neurological deterioration, *n* (%)	153 (51.5)	193 (57.1)	152 (52.6)	158 (54.7)	Risk ratio	0.90 [0.78 to 1.04]	0.159	0.96 [0.83 to 1.12]	0.617
					Risk difference (%)	−5.6 [−13.3 to 2.2]	0.158	−2.1 [−10.2 to 6.1]	0.617
*Safety outcomes*									
Post‐procedural ICH, *n* (%)	91 (30.6)	108 (32.0)	88 (30.4)	96 (33.2)	Risk ratio	0.96 [0.76 to 1.21]	0.722	0.92 [0.72 to 1.16]	0.475
					Risk difference (%)	−1.3 [−8.5 to 5.9]	0.722	−2.8 [−10.4 to 4.8]	0.475
Post‐procedural ICH category^c^					Chi‐square	—	0.419	—	0.179
HI 1, *n* (%)	28 (9.4)	25 (7.4)	27 (9.3)	21 (7.3)					
HI 2, *n* (%)	14 (4.7)	12 (3.6)	14 (4.8)	10 (3.5)					
PH 1, *n* (%)	23 (7.7)	37 (11.0)	22 (7.6)	37 (12.8)					
PH 2, *n* (%)	22 (7.4)	32 (9.5)	21 (7.3)	27 (9.3)					
Symptomatic ICH, *n* (%)^b^	41 (13.8)	52 (15.4)	39 (13.5)	46 (15.9)	Risk ratio	0.90 [0.62 to 1.31]	0.574	0.85 [0.57 to 1.26]	0.411
					Risk difference (%)	−1.6 [−7.0 to 3.9]	0.573	−2.4 [−8.2 to 3.4]	0.411
90‐day death, *n* (%)	70 (23.6)	80 (23.7)	68 (23.5)	73 (25.3)	Risk ratio	1.00 [0.75 to 1.32]	0.977	0.93 [0.70 to 1.25]	0.628
					Risk difference (%)	−0.1 [−6.7 to 6.5]	0.977	−1.7 [−8.7 to 5.3]	0.628

Abbreviations: CI, confidence interval; HI, hemorrhagic infarction; ICH, intracerebral hemorrhage; IQR, interquartile range; mRS, modified Rankin scale; NIHSS, National Institutes of Health Stroke Scale; PH, parenchymal hemorrhage; TICI, treatment in cerebral infarction.

^a^ TICI score was defined according to the Zaidat et al. modified treatment in cerebral infarction (mTICI) classification system.

^b^ Symptomatic hemorrhage was defined as any intracerebral hemorrhage associated with a ≥ 4 points worsening on the NIHSS score.

^c^ Any intracerebral hemorrhage was defined as any category according to the Heidelberg bleeding classification system.

### 3.2 Secondary Effectiveness Outcomes

Our analysis showed no significant differences in the rate of post‐procedural favorable TICI, post‐procedural TICI score distribution, 24‐h change in NIHSS score from baseline, 24‐h early neurological improvement, and 24‐h early neurological deterioration between women and men in the unmatched and matched cohorts (Table 2).

### 3.3 Safety Outcomes

There were no significant differences in the risk of any safety event between women and men in the unmatched and matched cohorts (Table 2).

### 3.4 Subgroup Analysis for the Primary Effectiveness Outcome in the Matched Cohort

**FIGURE 3:**
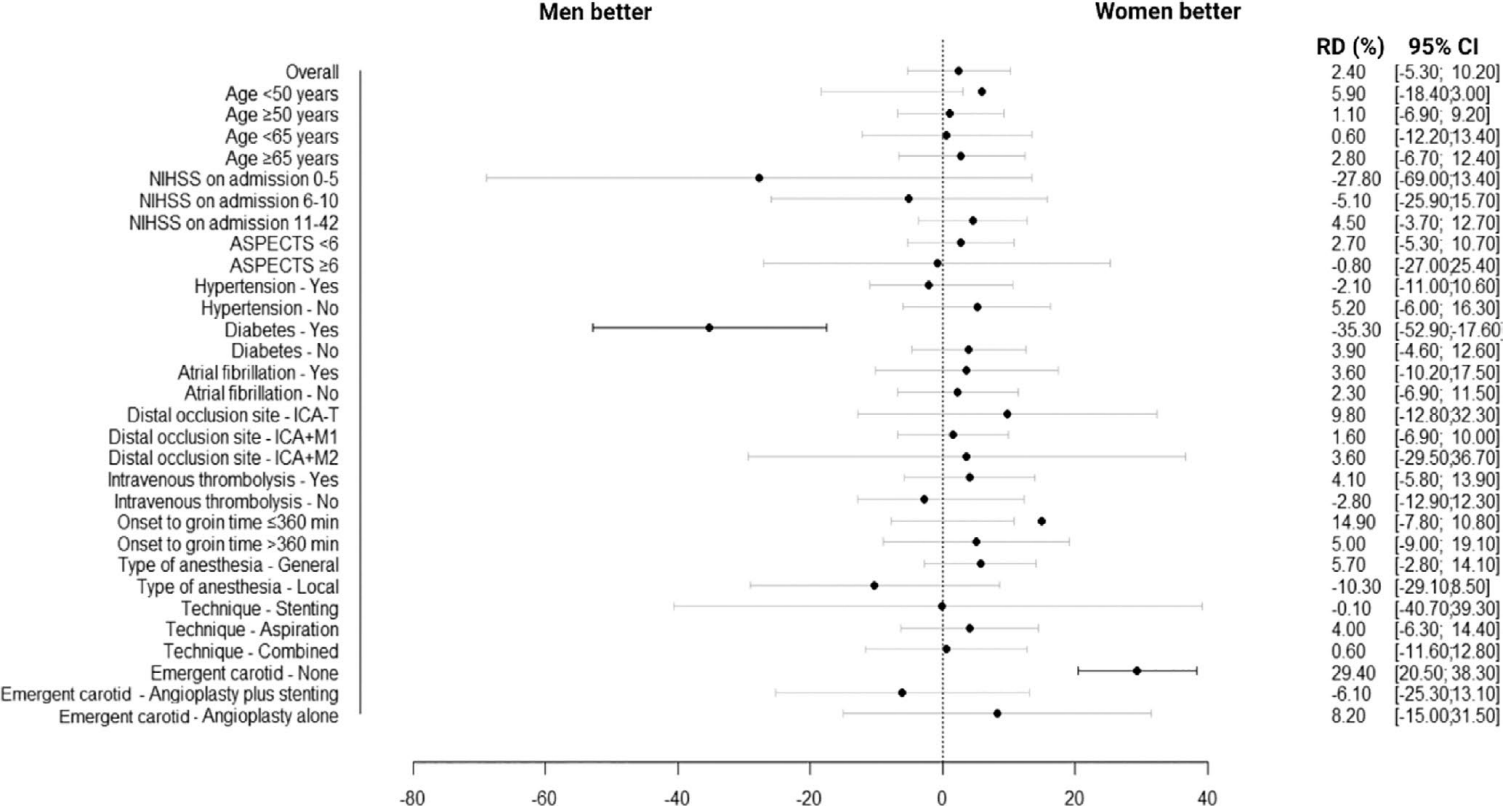
Subgroup analysis for the primary effectiveness outcome in the matched cohort. ASPECTS, Alberta Stroke Program Early CT score; CI, confidence interval; NIHSS, National Institute of Health Stroke Scale; RD, risk difference.

Results of subgroup analysis for the primary effectiveness outcome in the matched cohort are presented in Table 3 and Figure 3. Compared to men, we found a significantly lower rate of favorable 90‐day mRS score (0–2) in women with a history of diabetes (risk difference −35.3% [95% CI −52.9% to −17.6%]; *p* < 0.001). Moreover, we showed a higher rate of 90‐day favorable outcome post‐MT for anterior circulation tandem occlusion in women who did not receive any treatment for emergent carotid occlusion (risk difference 29.4% [95% CI 20.5 to 38.3]; *p* < 0.001). There was no significant risk difference between women and men across other subgroups, and we found no heterogeneity when testing the interaction between sex and each subgroup variable (Table 3).

**TABLE 3:** Primary effectiveness outcome by prespecified subgroups in the matched cohort.

	No. of patients	No. of patients with primary outcome/total no. (%)		Risk difference (%) [95% CI]	*p*	*p* value interaction
		Women	Men			
Overall	578	104/289 (36.0)	97/289 (33.6)	2.4 [−5.3 to 10.2]	0.541	—
Age, years						0.727
< 50	65	22/37 (59.5)	15/28 (53.6)	5.9 [−18.4 to 3.0]	0.635	—
≥ 50	513	82/252 (32.5)	82/261 (31.4)	1.1 [−6.9 to 9.2]	0.785	—
Age, years						0.753
< 65	230	52/120 (43.3)	47/110 (42.7)	0.6 [−12.2 to 13.4]	0.926	—
≥ 65	348	52/169 (30.8)	50/179 (27.9)	2.8 [−6.7 to 12.4]	0.561	—
NIHSS score on admission						0.972
0–5	19	2/9 (22.2)	5/10 (50.0)	−27.8 [−69.0 to 13.4]	0.168	—
6–10	90	20/38 (52.6)	30/52 (58.0)	−5.1 [−25.9 to 15.7]	0.633	—
11–42	469	77/242 (31.8)	62/227 (27.3)	4.5 [−3.7 to 12.7]	0.284	—
ASPECTS						0.323
< 6	47	4/16 (33.3)	8/31 (25.8)	2.7 [−5.3 to 10.7]	0.510	—
≥ 6	547	100/273 (36.6)	93/274 (33.9)	−0.8 [−27.0 to 25.4]	0.952	—
Hypertension						0.500
Yes	296	50/147 (34.0)	51/149 (34.2)	−2.1 [−11.0 to 10.6]	0.969	—
No	282	54/142 (38.0)	46/140 (32.9)	5.2 [−6.0 to 16.3]	0.363	—
Diabetes						0.413
Yes	108	16/53 (30.2)	36/55 (65.5)	−35.3 [−52.9 to −17.6]	**< 0.001**	**—**
No	470	88/236 (37.3)	78/234 (33.3)	3.9 [−4.6 to 12.6]	0.369	—
Atrial fibrillation						0.821
Yes	151	21/78 (26.9)	17/73 (23.3)	3.6 [−10.2 to 17.5]	0.606	
No	427	83/211 (39.3)	80/216 (37.0)	2.3 [−6.9 to 11.5]	0.625	
Occlusion site						0.806
ICA‐T	69	14/34 (41.2)	11/35 (31.4)	9.8 [−12.8 to 32.3]	0.398	
ICA + M1	474	81/239 (33.9)	76/235 (32.3)	1.6 [−6.9 to 10.0]	0.720	
ICA + M2	35	9/16 (56.3)	10/19 (52.6)	3.6 [−29.5 to 36.7]	0.830	
Intravenous thrombolysis						0.604
Yes	366	69/181 (38.1)	63/185 (34.1)	4.1 [−5.8 to 13.9]	0.418	
No	212	35/108 (32.4)	34/104 (32.7)	−2.8 [−12.9 to 12.3]	0.965	
Onset to groin time						0.660
≤ 360 min	415	77/206 (37.4)	75/209 (35.9)	14.9 [−7.8 to 10.8]	0.752	
> 360 min	163	27/83 (32.5)	22/80 (27.5)	5.0 [−9.0 to 19.1]	0.483	
Type of anesthesia						0.128
General	469	82/234 (35.0)	69/235 (29.4)	5.7 [−2.8 to 14.1]	0.187	
Local	109	22/53 (41.5)	28/54 (51.9)	−10.3 [−29.1 to 8.5]	0.281	
Distal thrombus thrombectomy technique						0.890
Stenting	24	5/11 (45.5)	6/13 (46.2)	−0.1 [−40.7 to 39.3]	0.973	
Aspiration	310	51/153 (33.3)	46/157 (29.3)	4.0 [−6.3 to 14.4]	0.444	
Combined	244	48/125 (38.4)	45/119 (37.8)	0.6 [−11.6 to 12.8]	0.925	
Treatment of emergent carotid						0.536
None	442	139/222	73/220	29.4 [20.5 to 38.3]	**< 0.001**	
Balloon angioplasty plus stenting	99	17/47	22/52	−6.1 [−25.3 to 13.1]	0.531	
Balloon angioplasty alone	37	4/20 (20.0)	2/17 (11.8)	8.2 [−15.0 to 31.5]	0.488	

*Note:* Statistically significant *p* values (< 0.05) are reported in bold.

Abbreviations: ASPECTS, Alberta Stroke Program Early CT score; CI, confidence interval; ICA, inner carotid artery; IQR, interquartile range; M, middle cerebral artery; mRS, modified Rankin Score; NIHSS, National Institutes of Health Stroke Scale; T, carotid T occlusion.

## 4 Discussion

In our propensity‐matched analysis of patients with anterior circulation tandem occlusion undergoing MT, overall, there was no difference between men and women in the primary, secondary effectiveness, and safety outcomes at 90 days following MT. However, women with diabetes had a worse outcome than their male counterparts. Furthermore, women treated with intracranial thrombectomy alone had a better 90‐day clinical outcome compared to men. No sex‐related differences were observed between women and men regarding other subgroups.

A recent meta‐analysis of seven randomized controlled trials on MT within the HERMES collaboration [20] showed that sex does not influence clinical outcome after MT. This analysis included 1762 patients, of whom 47% were women. However, it is noteworthy to mention that in this analysis of seven randomized controlled trials, the percentage of patients with anterior circulation tandem occlusion was less than 30%. Previous observational studies have investigated the prognostic factors associated with clinical and safety outcomes in patients with acute ischemic stroke due to tandem lesions of the anterior cerebral circulation with conflicting results regarding the impact of sex. Bracco et al. conducted a multicenter retrospective study including 227 patients with anterior circulation tandem occlusions consecutively treated with MT across five Italian stroke centers between 2015 and 2019 [9]. In this analysis, 67.8% of patients were men. On multivariate analysis, the authors showed that men were associated with a poor functional outcome. Conversely, subsequent observational studies that investigated clinical and procedural factors associated with clinical and safety outcomes in patients with tandem occlusions did not show a significant impact of sex on outcomes post MT for tandem occlusions. To date, our study is the first that used a propensity‐matched analysis to adjust for possible confounding factors to investigate the impact of sex differences on the clinical and safety outcomes of patients with anterior circulation tandem occlusion undergoing MT.

Another significant finding of our study is that diabetes might appear to affect outcomes after MT in patients with tandem occlusion differently by sex. According to our findings diabetes is more strongly associated with increased risk of 90‐day poor functional outcome in women compared to men post MT. Diabetes mellitus is an independent risk factor for stroke and cardiovascular disease [21]. Additionally, diabetes increases ischemic stroke severity and has been associated with poor functional outcome, worse long‐term vascular prognosis, and increased mortality after stroke onset [22, 23, 24]. However, whether diabetes can impact differently stroke outcome in women compared to men is still uncertain [25]. Findings from previous studies have been inconsistent, with some investigators reporting either a stronger or similar effect of diabetes on stroke outcome in women compared to men [24, 26, 27, 28]. However, it is notable to remark that these studies included patients with any acute ischemic stroke, and they were not specifically focused on patients undergoing MT for tandem occlusions. Moreover, many of these studies are unadjusted or adjusted only by age and vascular risk factors or previous treatments without contemplating two of the most important predictors of mortality: previous disability and stroke severity. Based on our findings, we could not clarify the reasons for these sex differences in outcome between diabetic women and men with tandem occlusion undergoing MT. While our study identified significant sex‐based differences in outcomes, particularly in diabetic patients, the underlying biological mechanisms remain unclear. Given the observational nature of this study, we were unable to explore pathophysiological factors such as hormonal, genetic, or metabolic differences that may influence stroke outcomes in women compared to men. Nevertheless, it is noteworthy to mention that diabetes is more strongly associated with worse post stroke clinical outcomes in women with diabetes [29, 30, 31] compared with men. Moreover, our findings of worse outcomes in diabetic women may be linked to sex‐specific metabolic and vascular differences observed in diabetes [32]. Previous studies suggest that women require greater metabolic deterioration to develop diabetes compared to men, including worse baseline levels of cholesterol, triglycerides, and blood pressure. Additionally, diabetic women exhibit greater endothelial dysfunction, dysregulated fibrinolysis, and a more pro‐thrombotic state, which diminish their natural vascular protection [33]. These mechanisms, potentially influenced by complex interactions between insulin and estrogen signaling, could contribute to the observed disparities in stroke outcomes [32, 33, 34]. Future research should focus on exploring these biological differences to better understand and address sex‐specific disparities in stroke outcomes, particularly in high‐risk subgroups such as patients with diabetes. In our study, 635 patients with tandem occlusion were analyzed, including 297 (46.8%) women, allowing a robust exploration of sex‐based differences through propensity score matching. While this near‐balanced representation is an improvement over prior stroke studies [35], the historical underrepresentation of women in clinical trials may still influence the generalizability of our findings. The observed worse outcomes in diabetic women compared to men highlight the need for further investigation into unexamined biological, metabolic, and treatment‐related factors. Enhancing female representation in future trials is essential to better understand and address sex‐specific responses to stroke interventions.

We observed a higher rate of 90‐day favorable outcome post‐MT for anterior circulation tandem occlusion in women treated with intracranial thrombectomy alone. Meta‐analyses of the previous observational studies comparing different treatment strategies reported conflicting results, while one meta‐analysis reported better outcome with cervical ICA stenting [36], one reported no difference in functional outcome [37], and another reported a higher complication rate and longer procedure time with cervical ICA stenting [38]. A recent pooled analysis from the TITAN and ETIS registries compared the functional and safety outcomes between different treatment approaches for the cervical ICA lesion during endovascular therapy for acute ischemic strokes due to tandem occlusion [39]. Patients treated with acute cervical ICA stenting for tandem occlusion strokes had higher odds of 90‐day favorable outcome, despite higher odds of intracerebral hemorrhage. While the subgroup analysis of this study demonstrated heterogeneity according to the lesion type, the relationship between sex and stent treatment for tandem occlusion strokes was not investigated [39]. In the absence of definitive findings from randomized controlled trials, our analysis has added further observational evidence not supporting a possible benefit of intracranial thrombectomy alone in female patients with tandem occlusion undergoing MT. However, the findings of our analysis should be interpreted with caution. Ongoing RCTs will provide more definite answers in the coming years, and will capture, beyond 90‐day outcomes, which subgroups of patients with tandem occlusion will benefit most from intracranial thrombectomy alone or stenting while undergoing MT.

Our analysis had the following strengths: (1) data ascertainment was undertaken systematically and rigorously in each registry from which the analyses were pooled; (2) the use of a propensity‐matched analysis to adjust for possible confounding factors; (3) overall large cohorts of patients. Nevertheless, our study had several limitations. First, the non‐randomized design of the study might have introduced bias. Even if we implemented a rigorous propensity score matching, we cannot exclude residual confounding due to the observational design of the study. Indeed, despite the power of propensity score matching, it should be noticed that potential unmeasured confounders may still have a role in driving treatment equipoise across sex groups. Further studies, ideally randomized controlled trials (RCTs), are required to confirm our findings and further reduce the risk of bias. Another possible limitation of our work is that we did not report competing stroke etiology as a variable in the propensity score analysis.

We acknowledge that the absence of detailed stroke etiology, particularly in patients referred from primary stroke centers, may have influenced our findings. This is because over 60% of the patients treated at our thrombectomy centers are referred from primary stroke centers. These patients are normally transferred back to the referring center 24 h after the procedure, and therefore we are not able to perform in these patients the basic investigations to establish any other competing etiology of their stroke. This limitation highlights the need for future studies to incorporate comprehensive etiological assessments to better understand their impact on outcomes. Finally, we also recognize the lack of standardized protocols for carotid treatment across centers as a limitation, which may have introduced variability in operator decision‐making and influenced the observed outcomes.

It is important to highlight that the findings from our subgroup analysis are exploratory and should be interpreted with caution, pending validation in larger clinical studies. While we observed significant differences in the risk of the primary effectiveness outcome, no significant heterogeneity was detected in the interaction between sex and the subgroup variables. This lack of heterogeneity is likely due to insufficient statistical power or high within‐group variability in our cohort, both of which are inherent limitations of our observational study design. However, we cannot rule out the possibility that unmeasured confounders may have contributed to the observed risk differences across subgroups.

In conclusion, our results provide real‐world insights into outcomes related to sex‐based differences in patients with anterior circulation tandem occlusion undergoing MT. Our analysis revealed that there was no difference between men and women in the primary, secondary effectiveness, and safety outcomes at 90 days following MT. There were sex‐specific differences in women with diabetes and treated with intracranial thrombectomy alone. Our findings underscore the importance of considering sex‐related factors when managing ischemic strokes due to tandem occlusion and highlight the need for more research to further explore mechanisms underlying these sex‐based differences, especially given the underrepresentation of women in clinical trials.

## Author Contributions

**Lucio D'Anna:** conceptualization, investigation, funding acquisition, writing – original draft, writing – review and editing, visualization, validation, methodology, software, formal analysis, project administration, resources, supervision, data curation. **Matteo Foschi:** conceptualization, investigation, funding acquisition, writing – original draft, writing – review and editing, visualization, validation, methodology, software, formal analysis, project administration, resources, supervision, data curation. **Mariarosaria Valente:** data curation, supervision, resources. **Liqun Zhang:** data curation, supervision, resources, writing – review and editing. **Simona Sacco:** data curation, supervision, writing – review and editing. **Raffaele Ornello:** data curation, supervision, writing – review and editing. **Nina Mansoor:** writing – review and editing, visualization. **Matthew Fallon:** writing – review and editing, visualization. **Adelaida Gartner Jaramillo:** visualization, writing – review and editing. **Massimo Sponza:** visualization, writing – review and editing. **Vladimir Gavrilovic:** visualization, writing – review and editing. **Kyriakos Lobotesis:** visualization, writing – review and editing. **Gian Luigi Gigli:** visualization, writing – review and editing, writing – original draft, supervision. **Soma Banerjee:** supervision, data curation, resources, project administration, visualization, writing – review and editing, writing – original draft. **Giovanni Merlino:** writing – original draft, writing – review and editing, visualization, validation, methodology, data curation.

## Conflicts of Interest

S.B. is a key opinion leader for RAPIDAI. All other authors have no conflicts of interest.

## Supporting information


Figure S1.

## Data Availability

The data that support the findings of this study are available on request from the corresponding author. The data are not publicly available due to privacy or ethical restrictions.
