# Supplementary material for: Impact of Sex on Clinical Outcomes of Tandem Occlusion in Acute Ischemic Stroke Patients Treated With Mechanical Thrombectomy. A Propensity‐Matched Analysis
Source: Eur J Neurol. 2025 Jan 13;32(1):e70044. doi: 10.1111/ene.70044 (PMC11726627; doi:10.1111/ene.70044)
Supplement: Supplementary file 1 — Figure S1. [file ENE-32-e70044-s001.docx]

**Supplementary Figure 1. Mirrored plot of covariate balance between the unmatched and matched cohorts**

**
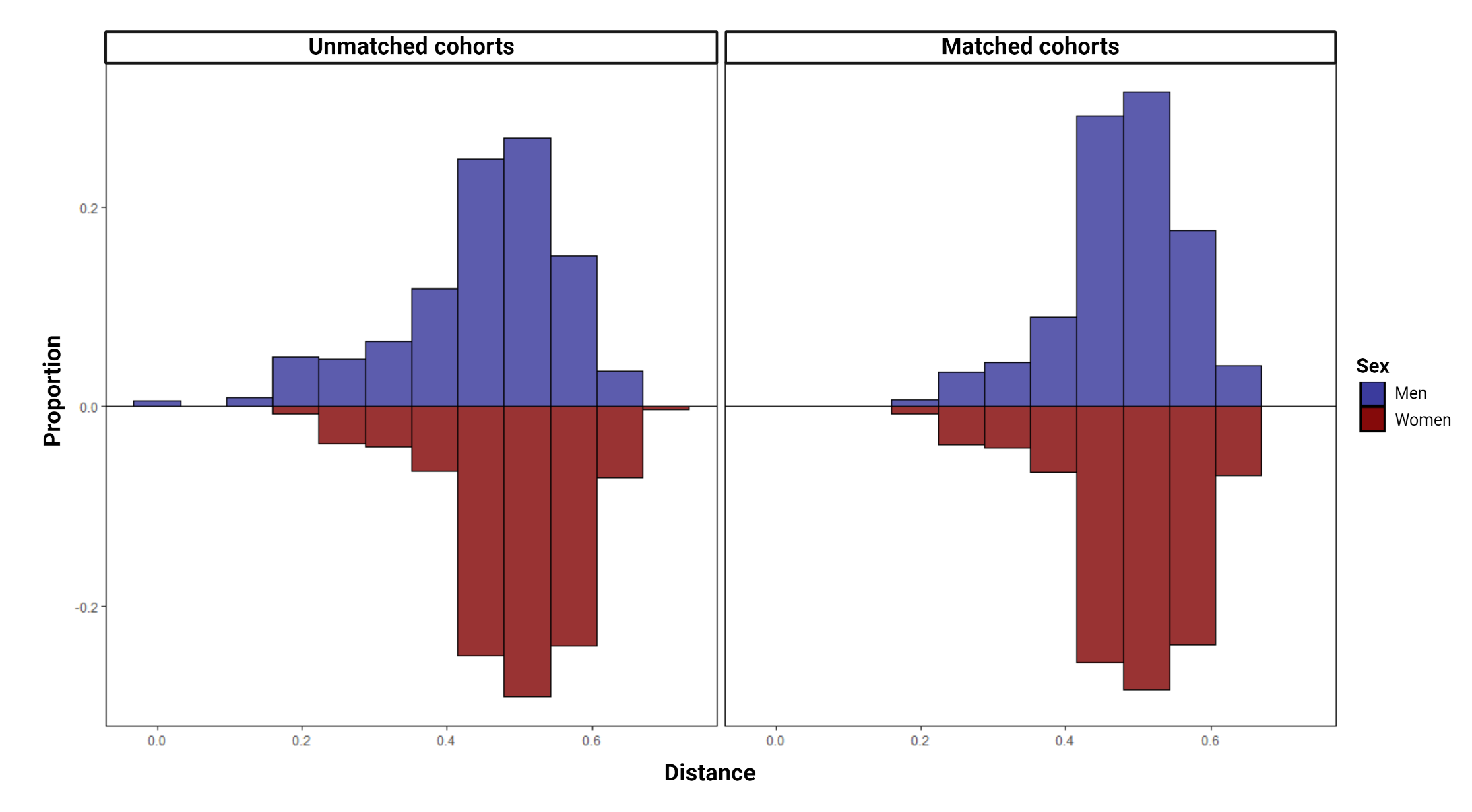
**

**Supplementary Figure 2. Love plot of covariate balance between the unmatched and matched cohorts**

**
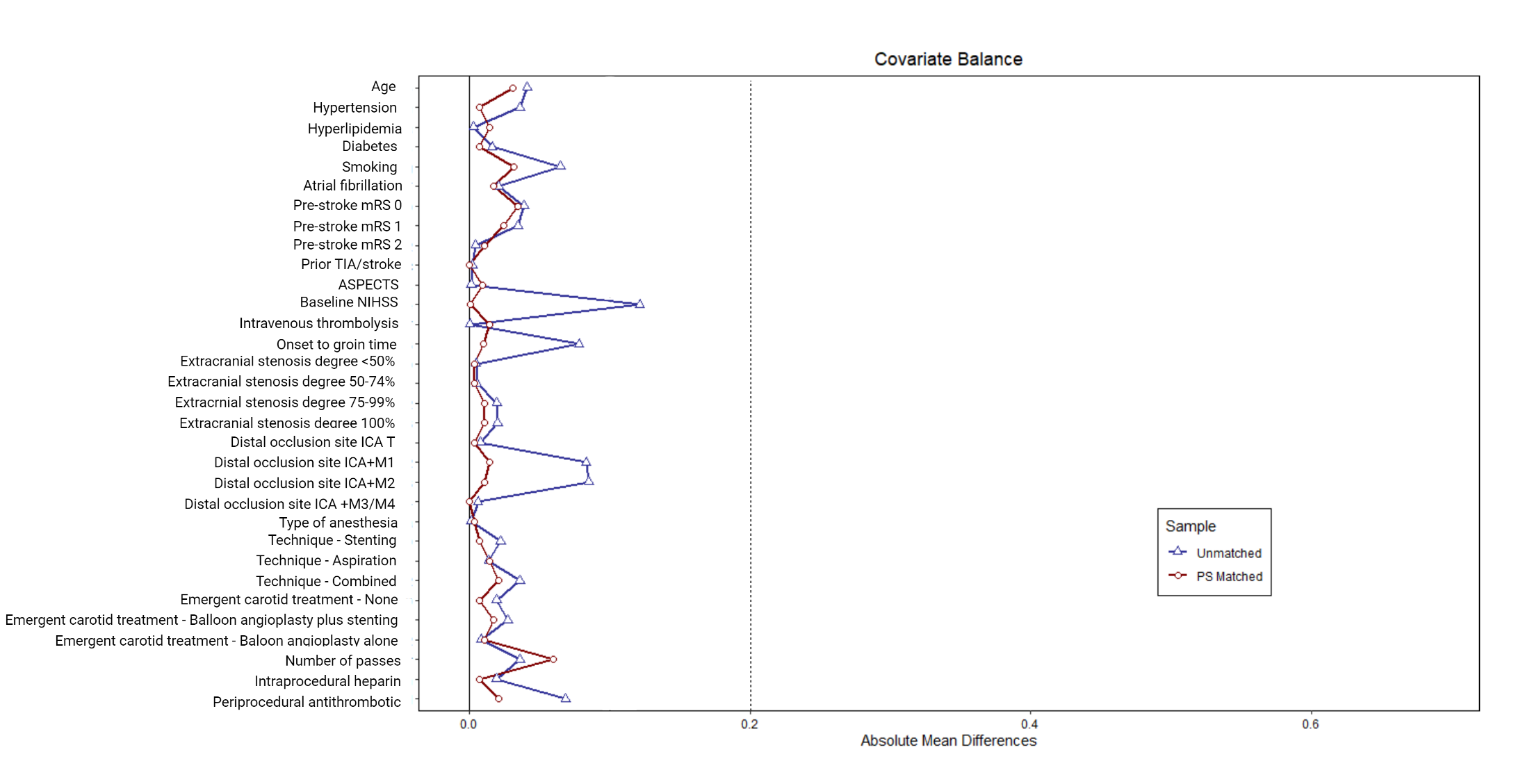
**

*Abbreviations. ASPECTS: Alberta Stroke Program Early CT score; mRS: modified Rankin score; ICA: inner carotid artery; M: middle cerebral artery; NIHSS: National Institute of Health Stroke Scale; PS: propensity score; T: carotid T occlusion.*
